# Biomarkers of related driver genes predict anti-tumor efficacy of immune checkpoint inhibitors

**DOI:** 10.3389/fimmu.2022.995785

**Published:** 2022-09-15

**Authors:** Shuai Jiang, Shuai Geng, Xinyu Luo, Can Zhang, Yang Yu, Mengfei Cheng, Shuo Zhang, Ning Shi, Mei Dong

**Affiliations:** ^1^ Department of Pharmacy, Harbin Medical University Cancer Hospital, Harbin, China; ^2^ Department of Pharmacy, Strategic Support Force Medical Center, Beijing, China; ^3^ Department of Ultrasound Diagnosis, Strategic Support Force Medical Center, Beijing, China

**Keywords:** cancer, immune checkpoint inhibitor, biomarker, overall survival, progress free survival

## Abstract

Cancer is a disease with high morbidity and mortality in the world. In the past, the main treatment methods for cancer patients were surgery, radiotherapy and chemotherapy. However, with early treatment, the recurrence rate of cancer is higher, and the drug resistance of cancer cells is faster. In recent years, with the discovery of immune escape mechanism of cancer cells, Immunotherapy, especially Immune Checkpoint Inhibitors (ICIs), has made a breakthrough in the treatment of solid tumors, significantly prolonging the overall survival time and disease-free progression in some solid tumors, and its clinical benefits are more prominent than those of traditional anti-tumor drugs, which has become the hope of cancer patients after the failure of multi-line therapy. More and more studies have shown that there is a correlation between cancer driving genes and the clinical benefits of ICIs treatment, and the therapeutic effects and adverse reactions of ICIs can be predicted by the status of driving genes. Therefore, screening potential biomarkers of people who may benefit from immunotherapy in order to maximize the therapeutic benefits is a top priority. This review systematically summarizes the cancer driving genes that may affect the clinical benefits of immune checkpoint inhibitors, and provides accurate scientific basis for clinical practice.

## Introduction

Tumor immunotherapy is a breakthrough research direction in the field of cancer therapy. It mainly inhibits and kills tumor cells by affecting the body’s immune system and enhancing anti-tumor immunity. This therapy has greatly changed the traditional tumor treatment strategy and brought more survival opportunities for patients (1, 2). Immune checkpoint inhibitors mainly include antibodies targeting cytotoxic T lymphocyte antigen-4 (CTLA-4) and antibodies targeting programmed cell death receptor-1 and its ligand (PD-1/PD-L1). CTLA-4 is a transmembrane protein, belonging to the immunoglobulin superfamily, which consists of extracellular domain, transmembrane domain and intracellular domain, and its extracellular domain is the receptor of B7 molecule (3). CTLA-4 competed with CD28 for binding to B7 ligand. Both CTLA-4 and CD28 molecules on the surface of T cells could bind to B7 ligand on the surface of antigen-presenting cell (APC), and the binding affinity of CTLA-4 was stronger than that of CD28. The binding of CD28 and B7 ligand produces synergistic stimulation signal, which can stimulate the activation of T cells and then produce the effect of killing tumor cells (4, 5). Contrary to the function of CD28, CTLA4 combined with B7 molecule to produce inhibitory signal, which blocked the effect of CD28 molecule on T cells, thus inhibiting the proliferation and activation of T cells (6). Programmed death receptor 1 (PD-1) is an important immunosuppressive molecule in CD28 superfamily, encoded by human PDCD1 gene, and its expression is enhanced under the stimulation of tumor necrosis factor. The main ligands of PD-1 are programmed death-ligand 1 (PD-L1) and PD-L2 (7). Immune checkpoint inhibitors can accurately occupy PD-1 or PD-L1 molecules, produce steric hindrance effect, hinder the binding of PD-1 and PD-L1, and restore immune responses inhibited by PD-1 pathway, including normal anti-tumor immune responses (8, 9). However, studies have shown that the inhibition rate of ICIs on solid tumors is only 10-40% (10). The results of this study show that a large number of patients do not benefit from immunotherapy. In addition, neo-antigen can also be recognized by T cells and cause immune response of tumor clearance. For example, the higher the tumor mutation burden (TMB), the neo-antigen, the higher the tumor immunogenicity and the higher the anti-tumor response of T cells. Therefore, there are individual differences in tumor types, ICIs types, susceptibility and new antigenicity of tumors, and biomarkers related to driving genes that determine the difference of clinical benefits of ICIs are the key to predict the curative effect of ICIs (11).

## Driver gene-related biomarkers

PD-L1 is an important immune checkpoint, which is called programmed cell death ligand 1 (PD-L1). PD-L1 antigen binding site is located in the variable region of Fab segment in the light chain of antibody structure, which determines the target of antibody and the target cells it acts on, while the constant region Fc segment of antibody structure determines the type of antibody, which binds to Fc receptor expressed by immune cells, resulting in antigen clearance (12). In current clinical practice, the expression intensity of PD-L1 is significantly correlated with OS and PFS of cancer patients after ICI treatment. The results of KEYNOTE 024 show that (13), compared with traditional chemotherapy drugs, OS and PFS treated with pembrolizumab are better for patients with advanced NSCLC with high expression of PD-L1 (≥ 50%), and when PD-L1 expression < 50%, the efficacy of immunotherapy is equivalent to that of traditional chemotherapy drugs. This indicates that the higher the expression level of PD-L1, the better the immunotherapy effect of NSCLC. The results of KEYNOTE-042 and CheckMate 227 showed that (14), compared with chemotherapy, the ICI group improved the overall survival time (OS) [Nivolumab plus ipilimumab: risk ratio (HR) 0.82, 95% CI 0.69-0.97; Pembrolizumab: (HR) 0.81, 95% ci 0.71-0.93]; In CheckMate 012 study (1), nivolumab combined with CTLA-4i ipilimumab was used to treat advanced NSCLC, and the effective rate of patients with PD-L1 ≥ 50% was over 90%. It exists not only in NSCLC, but also in other cancers. For example, triple negative breast cancer (TNBC) has a higher level of programmed cell death ligand 1 (PD-L1) expression, which is more likely to benefit from immune checkpoint treatment than other breast cancer subtypes. In 2019, according to the results of IMPASEN130 Phase III clinical trial (15), FDA accelerated the approval of atezolizumab combined with nab-paclitaxel to treat unresectable locally advanced or metastatic PD-L1 positive TNBC. In 2020, according to the results of KEYNOTE-355 Phase III clinical trial (16), FDA accelerated the approval of PD-1 inhibitor pembrolizumab combined with chemotherapy to treat locally relapsed, unresectable and metastatic PD-L1 positive TNBC. Therefore, PD-L1 positive subsets may benefit the most from immune checkpoint inhibitor (ICI) treatment, which can affect the therapeutic effect of clinical ICI to a certain extent.

### KRAS

RAS/Mitogen-activated protein kinase (MAPK) pathway plays a central role in the development of human cancer. It is highly activated in a variety of tumors, and many of its components have been identified as oncogene (17). The most common mutation of this pathway occurs in Kirsten rat sarcoma viral oncogene homologue (KRAS) (18). KRAS is a guanine nucleotide binding protein that regulates the mitogen-activated protein kinase pathway. When it is activated, it promotes downstream signal transduction and leads to cell growth and proliferation. In many cancers, KRAS mutation rate is high, such as 96% in pancreatic cancer, 52% in colorectal cancer and 32% in non-small cell lung cancer (19). KRAS mutant subtypes mainly include G12A, G12C, G12D, G12V and G13C. Up to now, although some targeted drugs are in clinical trials, they have not been approved to directly target the mutation of some subtypes of KRAS (20). At present, many studies have evaluated the influence of KRAS mutation on the curative effect of ICIs in cancer patients. A study on the prognostic characteristics and immunotherapy response of KRAS mutated non-squamous non-small cell lung cancer in East Asian population found that the disease remission rate (53.8% vs 8.3%, p = 0.030) and progression-free survival time (4.8 months vs 2.1 months, p = 0.028) of KRAS-non-G12C patients receiving ICIs treatment were higher than KRAS-non-G12C patients, and the tumor recurrence time of G12C patients (22.8 months) was shorter than that of KRAS-non-G12C patients (97.7 months, p = 0.004). For advanced NSCLC patients, there was a significant difference in OS between KRAS-G12C and KRAS-non-G12C patients (7.7 months vs 6.0 months, p = 0.018), while KRAS-G12V patients had the shortest OS (21). Another trial (22) retrospectively studied KRAS mutant non-small cell lung cancer patients treated with ICIs, suggesting mPFS (4.6 vs. 3.3 months) in KRAS mutant and non-KRAS mutant patients, but the results were not significant. Adi Kartolo et al. (23) evaluated the results of KRAS mutation in patients with advanced non-small cell lung cancer (NSCLC) with high expression of PD-L1 on treatment with first-line immune checkpoint inhibitors. The results showed that there was no significant difference in mOS between KRAS-MT and KRAS-WT patients (12.9 vs. 19.3 months, p = 0.879), and the trend of mOS deterioration in KRAS G12C patients was not significant compared with non-G12C and KRAS-WT patients (11.4 vs. 44.9, p = 0.772). In multivariate analysis, KRAS-MT status was independent of mOS (HR 0.901, 95% CI 0.417-1.946, p = 0.791). In patients with tumors with KRAS G12C variant treated with ICIs, the trend of declining survival rate is not significant. Therefore, KRAS mutation is positively correlated with the curative effect of ICIs in cancer patients, but KRAS-G12C mutation is correlated with the shorter tumor recurrence time in early NSCLC patients. Compared with KRAS-G12C, KRAS-G12V mutation is associated with shorter OS in patients with advanced NSCLC. However, it is worth noting that according to the summary analysis of ASCO FDA in 2022, the report shows that the status of KRAS has no effect on the tumor immune microenvironment of non-small cell lung cancer. The above related studies show that KRAS mutation is of great benefit to ICIs compared with KRAS WT patients. Therefore, we have reason to believe that the same driving genes may play different roles and functions in the formation of tumor immune microenvironment (TME) based on different solid tumors or genetic backgrounds.

### TP53

TP53 gene was first discovered in 1979 and is the first tumor suppressor gene to be discovered (24). Solid tumors are often accompanied by inactivation of TP53 function or pathway, which is related to the increase of malignant tumors, poor survival time of patients and drug resistance. This gene is involved in many biological processes, including DNA repair, cell cycle arrest, apoptosis, autophagy, metabolism and aging (25). The mutation rate of TP53 is high in cancers, and up to 50% of cancers contain two allele mutations of TP53 gene. TP53 gene has six most significant mutation sites, five of which are G to T mutations on codon containing methylated CpG sequence, including codon 157, 158, 245, 248 and 273 (26). Therefore, understanding the tumor-specific mutation profile of TP53 gene is very important for studying TP53-related carcinogenesis. A series of clinical studies have also been conducted to observe the effect of TP53 mutation on the clinical benefits of tumor patients treated with ICIs. Patient data obtained from a cancer genome map show (27) that TP53-MT is a potential indicator of relatively good response of bladder cancer patients to ICIs, and is related to prolonged overall survival (OS) [HR = 0.65 (95% CI 0.44-0.99), p = 0.041]. Through the comprehensive analysis of multiple platforms, it was found that TP53-MT patients showed stronger tumor antigenicity and tumor antigen presentation, higher tumor mutation load, higher new antigen load and higher MHC expression. Compared with TP53-WT, TP53-MT has stronger pre-existing anti-tumor immune effects in tumors, including interferon-γ enrichment, positive regulation of TNF secretion pathway and increased expression of some immunostimulating molecules (such as CXCL9 and CXCL10). Therefore, patients with TP53-MT are more likely to benefit from ICIs than patients with wild-type P53 (TP53-WT). As we know, tumor mutation burden (TMB) is related to tumor response to immune checkpoint inhibitors, and TP53 can also be used as an indirect quantification tool of tumor mutation burden (TMB). Sandra Assoun et al. (28) used next-generation sequencing to evaluate TP53 mutation in aNSCLC patients treated with programmed death-1 (PD-1) blockers. Tumor analysis of multiple TP53 mutations showed that patients with TP53 mutations had longer median OS (18.1 months vs. 8.1 months, p = 0.004), significantly longer median progression-free survival (4.5 months vs. 1.4 months, p=0.03), and higher objective remission rate (ORR) (51.2% vs. 20.7%, p=0.01). Xiangkun Wu et al. (29)discussed the relationship between TP53 mutation and immunophenotype of muscular invasive bladder cancer (MIBC) by comprehensively analyzing TP53 gene mutation and expression. A total of 99 differentially expressed immune-related genes (DEIGs) including ORM1, PTHLH and CTSE were identified based on TP53 mutation status, and the high-risk prognostic groups with poor prognosis were identified in The Cancer Genome Atlas (TCGA) and Gene Expression Omnibus (GEO) database. In addition, they showed lower expression of CD56 bright NK cells, CTLA4, LAG3, PDCD1, TIGIT and HAVCR2, and were more likely to respond to PD-1 and neoadjuvant chemotherapy than the low-risk prognosis group. Therefore, TIPS derived from TP53 mutation is a potential prognostic marker or therapeutic target, but additional prospective studies are needed to verify this potential marker.

### STK11/KEAP1

STK11 is a key upstream activator of AMP activated protein kinase and a central metabolic sensor, which participates in the response to intracellular energy changes through different cellular processes, including regulating glucose and lipid metabolism, cell growth and homeostasis (30). This genetic mutation of tumor suppressor gene leads to Peutz-Jeghers syndrome, which is a rare disease, which is characterized by easy development into benign and malignant tumors in different organ systems (31). In the mouse model of non-small cell lung cancer, STK11 mutation is related to “cold” immunosuppressive tumor microenvironment, showing a decrease in the expression of immune inflammatory factors (CD8+ and CD4+T lymphocytes, type 1 macrophages) and PD-L1, and an increase in T cell failure markers and tumor-promoting cytokines (32). STK11 mutation is more common in non-squamous NSCLC, with STK11 mutation occurring in 8-39% of patients (33). KEAP1 is the main regulator of nuclear factor erythroid-2-related factor-2 (NRF2, also known as NFE2L2), which plays a central role in cell response to oxidative stress and regulates the expression of a large number of genes. KEAP1 functional loss mutation occurs in about 11%-27% of NSCLC. KEAP1 mutation and NFR2 mutation are mutually exclusive, and are often related to simultaneous aberrations of targeted genes (such as 6% EGFR mutation and 18% MET amplification) and non-targeted genes (such as 45% TP53 mutation) (34). The absence of KEAP1-negative regulation determines the constitutive activation of NFR2, promotes tumor survival, and may also lead to drug resistance and poor prognosis of NSCLC patients (35). Biagio Ricciuti et al. (36)studied the relationship between STK11/keap1 mutation and KRAS mutation. The results suggest that in the joint cohort study involving 1261 patients, STK11 and KEAP1 mutations were associated with significantly worse progression-free (STK11 HR = 2.04, p < 0.0001; KEAP1 HR = 2.05, p < 0.0001) and overall(STK11 HR = 2.09, p < 0.0001; KEAP1 HR = 2.24, p < 0.0001) survival to immunotherapy uniquely among KRAS mut, but not KRAS wt LUADs. Gene expression ontology and immunocyte enrichment analysis showed that STK11 or KEAP1 mutation led to different immunophenotypes in KRAS mutation, but not in KRAS wild type and lung cancer. The results indicated that KRAS mutation status affected STK11/keap1 mutation and then affected the curative effect of ICIs. Simon Papillon et al. (35) studied the correlation between STK11 and KEAP1 and adverse reactions of immune checkpoint inhibitors. By analyzing the clinical and mutation data of 2276 patients, it is suggested that STK11 or KEAP1 mutation is related to poor prognosis in multiple therapeutic classes, while STK11 mutation is related to PFS treated with anti-PD-1/anti-PD-L1 (HR = 1.05; 95% CI 0.76-1.44; P=0.785) or OS (HR=1.13; 95% CI 0.76-1.67; P = 0.540). Similarly, KEAP1 mutation was also correlated with PFS (HR = 0. 93; 95% CI 0.67-1.28; P = 0.653) or OS (HR = 0.98; 95% CI 0.66-1.45; P = 0.913), which suggests that STK11/KEAP1 mutation is a prognostic marker rather than a predictive marker for anti-PD-1/anti-PD-L1 therapy. In another study (37), the prognostic effect of ICIs on patients with non-squamous non-small cell lung cancer (NSCLC) with STK11 or KEAP1 mutation was analyzed. Univariate and multivariate analysis showed that STK11/KEAP1 mutation was an independent and important prognostic factor affecting overall survival (P < 0.05) and progression-free survival (P < 0.05). Importantly, STK11/KEAP1 mutant patients showed poorer OS than wild type patients when receiving atezolizumab (all P < 0.05). In addition, for STK11 mutant subsets, atezolizumab did not improve OS (HR = 0.669; 95% Cl 0.380-1.179; P = 0.669), while the survival of KEAP1 mutation patients who received atezolizumab was improved (HR = 0.610; 95% Cl 0.384-0.969; P = 0.036).

### EGFR

Epidermal Growth Factor Receptor (EGFR) is a transmembrane glycoprotein and one of the four members of ErbB family of tyrosine kinase receptors. Activation of EGFR leads to autophosphorylation of receptor tyrosine kinase, which initiates a series of downstream signaling pathways involved in regulating cell proliferation, differentiation and survival. EGFR is abnormally activated through various mechanisms (such as receptor overexpression, mutation, ligand-dependent receptor dimerization, ligand independent activation, etc.), which is related to the occurrence of various human cancers (38). In cancer patients, while immune checkpoint inhibitors are used, EGFR status also provides a new treatment strategy for cancer patients, thus improving clinical outcomes. It is considered that the progress of tumor biology and tumor microenvironment (TME) differences in NSCLC with EGFR mutation may be a new method to enhance the curative effect of ICIs. Specific EGFR mutations affect the immunogenicity of TME and the response sensitivity to ICIs. Chen et al. (39) conducted a large-scale study on 600 EGFRm NSCLC patients in China. They reported that the OS of PD-L1 positive EGFRm NSCLC patients was worse than that of PD-L1 negative patients (median OS 15.2 vs 29.3 months, p = 0.006), although most of these patients also received EGFR TKI monotherapy in all treatment lines. Negrao et al. (40) reported that compared with patients with classical gene mutation, patients with metastatic EGFRm NSCLC benefited more from ICIs, ORR was 25% vs 0%, and disease control rate (DCR) was 50% vs 15%. Mazieres et al. (40, 41)analyzed the IMMUNOTARGET registry and compared the molecular characteristics of EGFRm patients’ response to ICIs. In this database, patients with EGFR exon 21 mutation had significantly longer PFS (2.5 months) than patients with EGFR exon 19 mutation (1.4 and 1.8 months, p < 0.001). Therefore, these studies indicate that EGFR mutation may increase the immunogenicity and immune response of ICIs. Future clinical trials should ensure that specific EGFR gene changes are reported and provide mutation subgroup data in order to further obtain evidence of this subject.

### MSI-H/dMMR

The main function of MMR is to correct the errors in DNA replication and ensure the fidelity of replication process. However, the hypermethylation and frameshift mutation of promoter lead to the loss of mismatch repair protein expression, which leads to MSI-H/dMMR. Patients with MSI-H/dMMR may benefit from PD-1/PD-L1 inhibitors, and about 15% of colorectal cancer patients have MSI-H gene test results (42). ASAOKA et al. (43) reported for the first time that 16 (57%) of 25 patients with MMR were treated with Pembrolizumab, and the other 9 patients (32%) were stable (SD). In 2017, Pembrolizumab became the first anti-PD-1 drug approved in the United States, suggesting that MMR status can predict the clinical efficacy of Pembrolizumab. HAUSE et al. (44) analyzed 5930 genomes of multiple tumors by genome sequencing, and found that MSI-H existed in 14 kinds of malignant tumors. The frequency of MSI-H in colorectal cancer, gastric cancer and endometrial cancer was significantly higher than that of other tumors, but the proportion of malignant tumor patients was still small. At present, it is generally recognized that patients with gastric and colorectal malignant tumors and MSI-H/dMMR in tumor tissues have better curative effect and higher benefit rate when using PD-1/PD-L1 inhibitor.

### HLA

Human leukocyte antigen (HLA) is the expression product of human major histocompatibility complex gene. HLA plays an important role in immune presentation and recognition. CD8+T cell-dependent killing requires human leukocyte antigen class I (HLA-I) molecules to present tumor antigens effectively. The loss of HLA diversity will lead to the decrease of immunotherapy response rate (45). Studies have shown that in patients with malignant melanoma and lung cancer, the A, B and C genes of HLA-I molecule are all heterozygous compared with patients with at least one gene homozygous, and the curative effect of immunotherapy is better; If all heterozygous patients have high TMB, the prognosis is better than patients with at least one gene homozygous and low TMB (46). HLA-B44 is a supersubtype of HLA, which can cross-present new antigens presented by other subtypes of HLA, which increases the diversity of HLA. Studies have shown that patients with HLA-B44 positive and high mutation level have higher survival rate (47).

## Discussion

This review explored the influence of driving genes on the therapeutic effect of ICIs, but the diversity and complexity of driving genes also have certain influence on tumor microenvironment. At present, it has been found that many immunotherapy markers are related to tumor microenvironment. For example, lymphocytes, macrophages and interstitial cells in tumor immune microenvironment also express PD-L1, and the expression level of PD-L1 also has certain influence on tumor microenvironment. For example, lymphocytes, macrophages and interstitial cells in tumor immune microenvironment also express PD-L1, and the expression level of PD-L1 also has certain influence on tumor microenvironment. In lung cancer, the level of PD-L1 was significantly correlated with the site of biopsy, with the highest expression in adrenal and liver metastases and the lowest expression in bone and brain metastases. At the same time, the level of PD-L1 in lung and distant metastatic tissues is positively correlated with clinical benefit, but the level of PD-L1 in lymph node metastasis may not be correlated with clinical benefit. Similar conditions exist in other driving genes, which suggest that driving genes have different roles in different tumor microenvironments.

Based on the above research and discussion, it is not difficult to find that the state of tumor driving genes affects the therapeutic effect of ICIs. However, it is more noteworthy that the influence of driving genes on the immune microenvironment of different tumors determines the predicted value of ICIs. As shown in Meichen Gu et al. (48), KRAS/LKB1 and KRAS/TP53 common mutations produce different immune signals in lung adenocarcinoma. New data suggests that KRAS-mutated lung adenocarcinoma can exhibit enhanced PD-L1 expression and additional somatic mutations, linking the prospect of immune checkpoint blockade therapy being applied to the disease. However, the response of lung adenocarcinoma with kras mutation to this treatment is different, which is largely attributed to the heterogeneity of tumor immune environment. Recently, it has been found that lung adenocarcinoma with KRAS-mutation expresses LKB1 or TP53 mutation at the same time, and its tumor immune characteristics are usually different. Tumors with KRAS/TP53 co-mutation usually have significant up-regulation of PD-L1 expression and accumulation of tumorigenic t cells, while tumors with KRAS/LKB1 co-mutation usually have negative PD-L1 expression and few tumorigenic immune infiltration. Therefore, in addition to PD-L1 expression, detection of TP53 or LKB1 mutation will hopefully guide the clinical use of immune checkpoint blocking therapy for kras mutant lung adenocarcinoma.

Tumor formation is the result of immune escape, and ICIs can reverse immune escape and restore the body’s ability to recognize and eliminate tumor cells. Immunotherapy opens up a new model of cancer treatment. The biomarkers that predict the cancer efficacy, adverse reactions and drug resistance of ICIs play an important role in screening ICIs beneficiaries. Among them, efficacy markers PD-L1, TMB and MSI/MMR have entered the guidelines or consensus, while there are few studies on other driving gene markers of immunotherapy, such as EGFR, HLA, TP53, etc., and the exploration of more accurate biomarkers is still the focus of research. In addition, more related biomarkers and other factors affecting survival and prognosis of immunotherapy (such as tumor microenvironment, intestinal flora, DNA repair damage, etc.) need to be further explored and studied. At present, there are still few large sample trial data based on Chinese population, but with the continuous development of cancer ICIs clinical trials and the increasing number of treatment cases, it is believed that tumor markers will play an increasingly important role in predicting the efficacy, survival prediction and adverse reactions of ICIs. At the same time as the specification of biological detection technology, progress of gene diagnosis technology and medical data, the rapid development of new technologies and means such as artificial intelligence, immunotherapy of cancer will shift from illness condition as they intend and, since the future is expected to be through the detection of biomarkers to predict treatment in patients with different stages of treatment benefits and risks, In this way, precise and individualized treatment plans can be developed to enable patients to have a longer survival time and a higher quality of life. This will be our next research direction.

At the same time, it has become the focus of clinical research to explore new and different combination therapy modes and improve the immunotherapy response rate. Combination therapy can overcome the limitations of monotherapy. ICIs has elicited a lasting clinical response in some patients, which is largely dependent on effective T cell infiltration and effector T cell function in TME, while combination therapy is recommended to target multiple abnormalities in the differentiation of cancer cells and normal cells. It mainly includes decreasing TMB and enhancing tumor immunogenicity (such as in combination with chemotherapy, radiotherapy and targeted therapy), enhancing T cell transport and enhancing T cell response. The status of driver genes in cancer cells and normal cells will also provide better strategies for drug combination. In the future, with the progress of genomics, transcriptomics and immunodetection technology, the combination therapy with multiple ICIs will be a new development trend. The establishment of comprehensive biomarker evaluation system through bioinformatics and other methods can predict the efficacy of ICIs more comprehensively, thus promoting the development of tumor precision medicine.

### Search strategy and selection criteria

**Figure 1:**
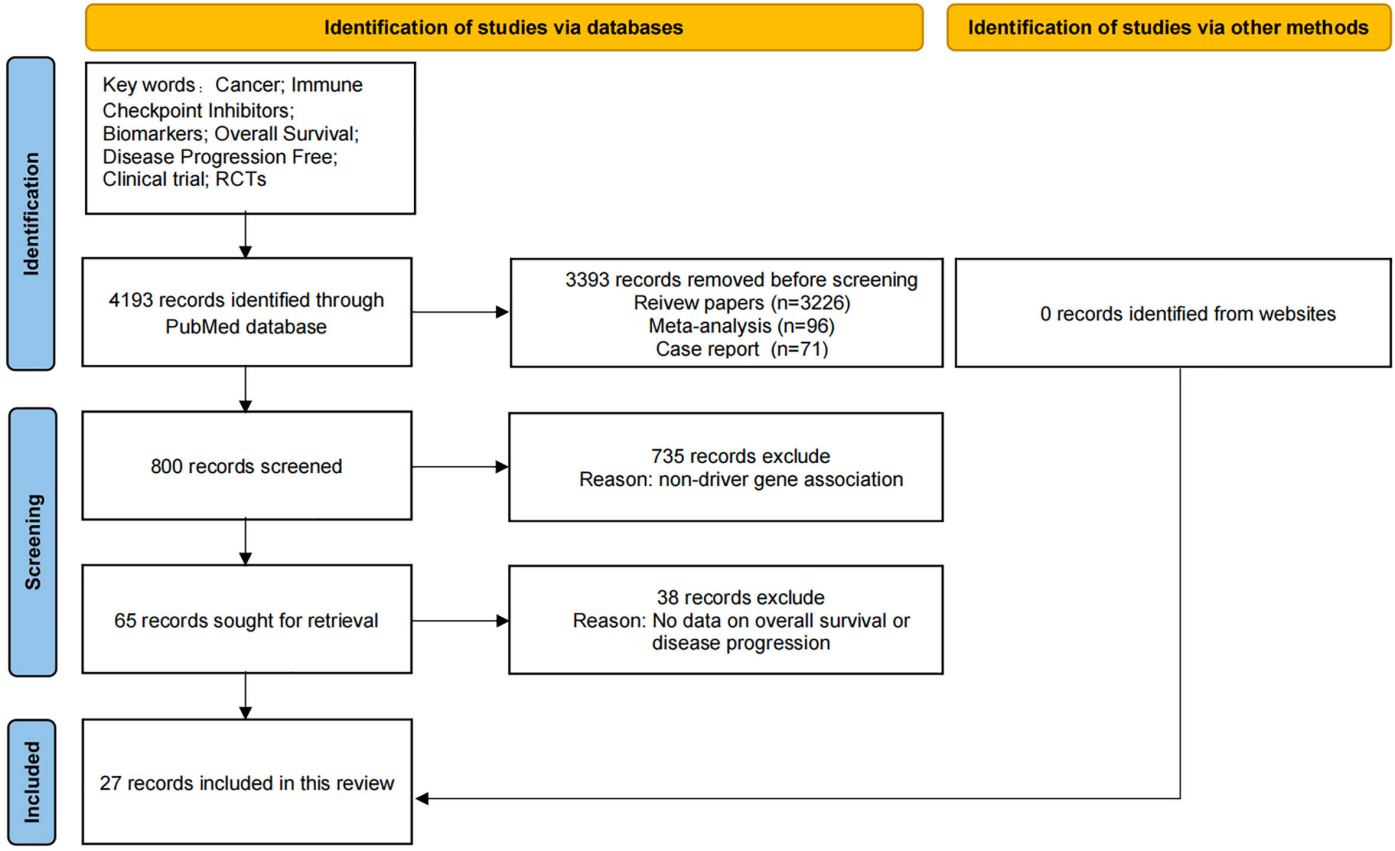
PRISMA Flow chart of article selection.

As shown in **Figure 1**, the data for this review was obtained by searching PubMed with key words “cancer; Immune checkpoint inhibitors; Biomarkers; Overall survival; No disease progression “retrieved from related articles. We identified 4193 records through PubMed database search, but did not find relevant information records through other sources. Before screening, we deleted 3393 literatures, including records of review literatures (n = 3226), meta-analysis (n = 96), and case reports (n = 71). In addition, 735 references without relevant driver gene introduction were excluded. Another 38 literatures without relevant data such as OS and PFS were excluded. Finally, the review included 27 records. Only articles published in English between 2000 and 2022 are included.

## Author contributions

SJ and SG collected data and wrote the paper. XL, CZ, YY, MC and SZ collect literature and information. NS and MD reviewed the paper. All authors read and approved the final manuscript.

## Funding

This work was supported by Beijing Hongdingxiang Public Welfare Development Center (BJ-HDX-20220437) and Project of Beijing Medical Award Foundation (YXJL-2022-0187-0013).

## Conflict of interest

The authors declare that the research was conducted in the absence of any commercial or financial relationships that could be construed as a potential conflict of interest.

## Publisher’s note

All claims expressed in this article are solely those of the authors and do not necessarily represent those of their affiliated organizations, or those of the publisher, the editors and the reviewers. Any product that may be evaluated in this article, or claim that may be made by its manufacturer, is not guaranteed or endorsed by the publisher.
